# Creating Inquiry Between Technology Developers and Civil Society Actors: Learning from Experiences Around Nanotechnology

**DOI:** 10.1007/s11948-015-9660-2

**Published:** 2015-06-04

**Authors:** Lotte Krabbenborg

**Affiliations:** Institute for Science, Innovation and Society (ISIS), Radboud University, Heyendaalseweg 135, P.O. Box 9010, 6500 GL Nijmegen, The Netherlands

**Keywords:** Upstream public engagement, Dewey, Emerging technologies, Reflective inquiry, Civil society actors, Dialogical governance

## Abstract

Engaging civil society actors as knowledgeable dialogue partners in the development and governance of emerging technologies is a new challenge. The starting point of this paper is the observation that the design and orchestration of current organized interaction events shows limitations, particularly in the articulation of issues and in learning how to address the indeterminacies that go with emerging technologies. This paper uses Dewey’s notion of ‘publics’ and ‘reflective inquiry’ to outline ways of doing better and to develop requirements for a more productive involvement of civil society actors. By studying four novel spaces for interaction in the domain of nanotechnology, this paper examines whether and how elements of Dewey’s thought are visible and under what conditions. One of the main findings is that, in our society, special efforts are needed in order for technology developers and civil society actors to engage in a joint inquiry on emerging nanotechnology. Third persons, like social scientists and philosophers, play a role in this respect in addition to external input such as empirically informed scenarios and somewhat protected spaces.

## Introduction

The development of newly emerging sciences and technologies (NEST) has for long been a prerogative of science and industry, with increasing attempts of government agencies to nudge and perhaps steer. Nowadays, citizens and civil society organizations (CSOs) are expected to participate as new dialogue partners, already during the early stages of the technology development, including the R&D phase (European Commission 2002, 2011; Wilsdon and Willis 2004).

The participation of civil society actors is particularly visible in the development of newly emerging nanosciences and nanotechnologies where early, so-called upstream public engagement has been piloted in a number of countries and in different ways, ranging from one-day events (for example, see Katz et al. 2009; Van Oudheusden and De Zutter 2012) to fully fledged societal dialogues (Pfersdorf 2012; Krabbenborg 2012).

Actually, upstream public engagement is part of a larger effort to create anticipatory governance of nanotechnology. Instead of waiting for societal impacts to become visible in society, government agencies and some technology developers are now trying to anticipate possible societal impacts with the aim to make better, that is, more informed, decisions about the further development of nanotechnology in the here and now. Civil society actors are involved because they are expected to be knowledgeable in giving voice to concerns, needs, and wishes of society (European Commission 2001). The idea is that technology developers can become more responsive to societal needs and issues, and include these in their decision-making processes.

The early involvement of citizens and civil society organizations is to be welcomed because of the opportunities it provides to bring new perspectives, experiences and dilemmas into deliberation processes. However, more can be achieved. Rather than just calling for more participation (phrases such as ‘voices of civil society must be heard’ and ‘nanotechnology developers should engage with civil society’ testify to this), the open-ended character of newly emerging science and technology, that is, how to address the combination of promises and uncertainty of actual outcomes, have to be taken into account more explicitly in deciding when, how, and with whom to create early stage interaction initiatives. This now happens to some extent, but in general there is a division of labour between technology developers and civil society actors that creates limitations to articulation and learning on emerging technologies.

Limitations of the division of labour are very clear in the emphasis from the side of technology developers on providing factual knowledge on the new technology. This has been noted before (see for instance Te Molder 2011; Bickerstaff et al. 2010; Hanssen 2009; Ross 2007; Irwin 2006; Powell and Colin 2008). The other side of the division of labour then is that civil society actors are expected to recognize and put forward societal needs and concerns (cf. the perception of non-governmental organizations (NGOs) as ‘voices of civil society’). Such a division of labour simplifies and underestimates the socio-technical complexity. It assumes that the emerging technology, as well as societal impacts, are already given, while in fact, in the early stages, newly emerging science and technology and its consequences are yet indeterminate. Promises are made, for instance about better food or cheaper healthcare, but nobody really knows what forms the technology will take, how it will materialize in society and what the societal impact might be (Barben et al. 2007; Stirling 2008). This implies that in the early stages societal issues are not just ‘out there’ and recognizable by civil society actors, but co-evolve with innovation trajectories.

For instance, the choices made by government or funding agencies with regard to sponsoring certain science and technology developments influence which promises can be further developed by scientists working at universities or companies. In turn, the activities of scientists or industrialists—for example to develop certain functionalities in medical devices—influence the actions of users. In general, the activities of different groups of actors are entangled one way or another. Together, they add up to emerging patterns of how a new technology can materialize in society and what the societal impacts might be. Thus, acquiring insight into societal issues pertaining emerging technologies is not just a matter of including civil society actors as new dialogue partner because of their presumed predisposition to be knowledgeable about societal issues and needs. Instead, it is a joint effort of all actors involved. Because of the co-evolution between technology and society, the question of what is at stake and for whom must all be inquired into and further articulated. To do so, all actors involved in an innovation trajectory should, ideally, share and discuss their activities and considerations as these influence the type of societal issues that emerge.

The challenge is thus how to overcome the simple distribution of roles and responsibilities between technology developers and civil society actors in order to address the open-ended character of NEST more productively. The philosopher John Dewey (1859–1952) developed an interesting approach. The aim of this paper is to first discuss Dewey’s thought on public engagement in relation to new science and technology and second, to examine, by studying four novel spaces for interaction in the domain of nanotechnology, whether and how elements of Dewey’s thought are visible and under which conditions. I will conclude by briefly reflecting upon the question how Dewey’s thought on public engagement can be productive for ongoing policy attempts to improve public and stakeholder engagement in innovation trajectories (cf. European Commission 2014).

## Publics Emerge and Disappear

For Dewey, there is no public or civil society that exists beforehand with which stakeholders—in our case, developers or promotors of nanotechnology—can engage. A public always emerges in relation to concerns about an indeterminate situation: a situation in which it is not clear how to act, what to value, and what is at stake. An example of indeterminacy is how the further development of telephony in the time of Dewey destabilized the way people were associating and communicating. Due to telephony people could maintain relations at a distance without actually meeting each other. However, in everyday interpretative frameworks (values, norms, roles, and responsibilities), small-scale communities and face-to-face contact remained the main point of reference. These existing interpretative frameworks, inherited from a different period, were not necessarily adequate to grasp new realities, and led to ‘uneasy equilibriums’ between the old and the new (Dewey 1920, p. XXXII).

For Dewey, a public consists of those who are involved in an indeterminate situation, or to be more precise, Dewey states that when the consequences of acts, such as the development and introduction of new science and technology, ‘extend beyond those directly involved, and affect the welfare of many others, the act acquires a public capacity’ (Dewey 1927, p. 13). In other words, when those who were not involved in an act are affected by it in such a way that existing institutions and arrangements fail to address the consequences adequately and indeterminacy is experienced, then publics emerge. As such, publics do not have an a priori social, geographical, or institutional status, but they are bound by an issue (Marres 2005, 2007). Dewey scholar Brown (2009) argues that the term public has an active as well as passive connotation for Dewey. Those who are indirectly affected form a ‘passive protopublic’. The active element is that a public, ideally, should organize itself and start a reflective inquiry.^1^ A reflective inquiry refers to a cooperative and communicative investigation between those who experience indeterminacy, thereby discovering—by inquiry, imagination and experimentation: what are the issues; who is involved; which ends to pursue and what to value? (Dewey 1920; Keulartz et al. 2004).

A reflective inquiry can be broken down into four successive phases (Dewey 1938, pp. 107–112; Hildebrand 2008, pp. 53-56). After the initial experience and recognition of an indeterminate situation, the first phase is to transform an indeterminate situation into a problematic one by means of an inquiry into and articulation of problems. Problems can be articulated by participants sharing experiences of how consequences of an act affect daily lives and routines. Dewey emphasizes that problems do not exist prior to an inquiry. In judging that it is a problem, we define it (Hildebrand 2008). In the second phase, participants should formulate hypotheses about possible solutions of how to deal with the problems. This interaction takes the form of forecasting, backcasting, and imagining the possible consequences of choosing a particular line of action. The third phase consists of an actual rehearsal of how new lines of action could work out in practice. In this phase, an estimate of possible consequences is made for those who are involved. The final phase of a reflective inquiry is the experimental testing or piloting (in real life) of the hypothesis that participants pointed out as the preferred solution. Dewey stresses that a solution should address as many issues as possible that were discovered during the inquiry process (Dewey 1957). Furthermore, he emphasizes that reflective inquiry should take the form of interaction and participation in the light of the unknown. What to value, how to act, and which ends to pursue emerge through sharing experiences and questioning each other. As such, a reflective inquiry refers to a space for interaction that functions as a way to articulate what is happening and to construct new or adapted interpretative frameworks through which an understanding of the new can be acquired. Moreover, such a space for interaction will make attempts to purposefully steer or modify what is happening more reflexively.^2^

The product of a reflective inquiry is situated and time-bound knowledge. Its value can be measured by whether it is able to solve actual problems, that is, whether it can ‘effect a working connection between old habits, customs, institutions, beliefs and new conditions’ (Dewey 2008a, p. 137).

Although Dewey elaborates the formation and activities of publics in more detail, I can limit myself at this stage to reiterate that Dewey‘s notion of ‘publics’ is not similar to contemporary notions of ‘civil society’ or the ‘general public’. In upstream public engagement events, the public is seen as a collective in its own right, and distinct from actors who see themselves responsible for the development and embedding of nanotechnology. In Dewey’s philosophy, there is no public beforehand that can be engaged with. Publics emerge in relation to an indeterminate situation and disappear when their business, that is, reflective inquiry, is concluded (Dewey 2008b, 10:23). With regard to emerging nanotechnology, this would imply that both nanotechnology developers and civil society actors, in principle, are part of the same public, as both groups of actors are implied in indeterminate situations occasioned by the development of nanotechnology, albeit in different ways, and with different roles and mandates. There is, for instance, an asymmetry in agency as technology developers have mandates to decide whether or not to take up the concerns, values, and wishes of civil society in their decision-making processes.

In the next sections I will explore to what extent elements of joint inquiry between nanotechnology developers and civil society actors already occur and under what conditions.

## Design of the Study

Four spaces for interaction were selected for examination, each chosen because something is visible of the ideal scenario of both nanotechnology developers and civil society actors sharing their views and concerns, jointly inquiring into an indeterminate situation and articulate what is at stake and what should be done. Let’s call this a reflective inquiry about NEST in the twenty-first century. We cannot expect to find a fully-fledged case of reflective inquiry, but we will be able to learn about various aspects, for instance about the formation of a ‘Deweyan public’, or the content of the problems that were articulated by the participants. The final choice for the four spaces was also guided by a practical consideration concerning data collection: a prerequisite to trace elements of reflective inquiry was that, as an analyst, I should have access to the space for interaction. Ideally I would be able to be a participant observer or otherwise capture the inquiry process in retrospect by interviewing participants and document analysis.

The four spaces for interaction that were selected are:

1. The partnership between the chemical company DuPont and a non-governmental organization Environmental Defense Fund (EDF). Between 2006 and 2009 these two actors collaborated with the aim to develop a joint risk framework for the development, use and disposal of engineered nanoscale materials (DuPont and Environmental Defense Fund). Developers and NGOs traditionally operate quite separately regarding the development of a new technology, but here they co-produced a risk framework.
2. A European Commission funded capacity building project, NanoCap (2006–2009), for civil society organizations. The consortium, consisting of four environmental NGOs, five trade unions and five universities, aimed to equip NGOs and trade unions with capacities to take up a new role as dialogue partner of science and industry within the development of nanotechnology. Meetings and working conferences with academic researchers and companies were part of the project.
3. The Dutch Societal Dialogue on nanotechnology (2009–2011). The Societal Dialogue was an attempt by the Dutch government to open up its policymaking on NEST by stimulating stakeholders and interested citizens to inquire into and articulate societal issues. The detailed design of the spaces for assembly was delegated to a Committee. The Committee in its turn was not part of actual interactions, but stimulated various distributed activities by funding bottom-up proposals for interaction activities.
4. Constructive Technology Assessment (CTA) workshops (2010) organized by social scientists within the domain of nanotechnology. In these workshops, technology developers and civil society actors could meet and in interaction inquire into real-world indeterminate situations.

The empirical data presented in this article are part of a larger study on the involvement of civil society actors in emerging technologies (Krabbenborg 2013a). This larger study provides detailed descriptions of the four case studies. This paper goes immediately into presenting elements that are important to flesh out conditions and proceedings of reflective inquiry about NEST in the twenty-first century.

## Nanotechnology Developers and Civil Society Actors Forming a Deweyan Public

A common characteristic for all the four cases was that special efforts were needed in order for nanotechnology developers and civil society actors to see themselves as embedded in an emerging indeterminate situation and engage in a reflective inquiry. Dewey developed his thinking in relation to existing technologies that were already part of a common world and a lived experience in people’s daily lives. As such, people could actually experience indeterminacy in their life, for instance because new technologies made it impossible for them to just continue in their daily routines. Newly emerging nanosciences and nanotechnologies are mainly hopeful promises and expectations, and do not yet exist as concrete products and systems embedded in a common world where society at large faces concrete dilemmas and issues. A consequence is that civil society actors might not be motivated to have nanotechnology as a topic for deliberation and reflection as there is nothing at stake for them. This was actually the case with the EU funded NanoCap project. For most of the environmental organizations and trade unions participating in NanoCap, the development and governance of nanotechnology was not a matter of concern. The civil society organizations (CSOs) took part in the project because they were invited to participate. The umbrella organization of Mediterranean environmental organizations (MIO-ESCDE) expressed interest in participating, but was concerned that only a very small number of the members they represented would be interested in the topic because there was as yet no nano-discussion in Mediterranean countries. Nevertheless, MIO-ESCDE finally decided to accept the invitation and over time its members welcomed the NanoCap project (email communication with author 05-09-2012). Thus, whereas for Dewey it is the existential experience of indeterminacy that leads to the identification of publics, to have emerging technologies as a topic for deliberation, one has to anticipate emerging indeterminate situations in order to point out who is involved.

However, owing to the way emerging technologies are developed in our society, it is relatively difficult for actors responsible for the development and embedding of new technologies to see how they are involved in an emerging indeterminate situation and with whom.

Tasks and mandates to develop new science and technology are distributed among various groups of actors (Garud and Ahlstrom 1997). Scientists and industrialists, for example, have a cultural mandate to work towards knowledge production and economic prosperity, while other actors, such as government agencies, are tasked with oversight and regulation. The work of these different actors is entangled and there is mutual influence, e.g. decisions taken by funding agencies influence the type of activities that can be carried out in laboratories. But at the same time actors also work in relatively “separate worlds.” One can be a good scientist and contribute to progress in science by writing papers about the technical details of nano-enabled drug delivery systems and never actually meet people who are expected to use these new systems, like physicians and patients. In our modern differentiated societies it is therefore difficult for actors to interact and become acquainted with one another and see how they are involved in an emerging indeterminate situation and with whom.

The cases demonstrated that ‘third persons’, i.e. persons working at the interfaces of science, society and policy can play a role in providing a first diagnosis of indeterminacy (Cf. Chilvers 2013). In the case of CTA workshops for example, analysts, such as social scientists or philosophers, acted as mediators between the different worlds. As already argued in Krabbenborg 2013b: a CTA analyst has a different position within an innovation trajectory. He or she can move around in the different worlds, observe, ask questions and study what is happening. Therefore, he or she can see things and point out issues, such as emerging indeterminate situations, that are more difficult to see for actors operating in a particular domain and subjected to realising their daily tasks and mandates properly (Krabbenborg 2013b, p. 184). Based upon a first articulation of an emerging indeterminate situation a CTA analyst can identify a Deweyan protopublic and organize a dedicated ‘bridging event’ to discuss the indeterminate situation.

Third persons also played a role in the Dutch Societal Dialogue. In total thirty-five projects were honoured funding to organize actual interaction events on nanotechnology. Twenty-eight of these projects were organized by intermediaries with a professional background in bridging technology and society, either as a science communicator (e.g. for magazines, museums, educational settings or science cafés), or as a researcher in the field of science and technology studies (www.nanopodium.nl; Hanssen et al. 2011).

In the case of DuPont and EDF an indeterminate situation was already on the agenda, namely the question if and how engineered nanoscale materials might pose health and environmental problems to society at large. EDF already explored nano-issues within their own organization and DuPont discussed risk issues with other stakeholders, for example through participation in ICON, a multi-stakeholder organization focused on nanotechnology and risk reduction that included representatives of large and small corporations, government agencies, academic institutions and non-governmental groups from around the world (www.icon.rice.edu). During the partnership of DuPont and EDF, the emphasis was on the further articulation and negotiation of what should be included in the risk framework (see also Krabbenborg 2013c). In order to stimulate interactivity, DuPont and EDF created a somewhat protected space. DuPont’s production processes for nanoscale materials were taken as a source for the inquiry into the health and environmental safety issues. As a DuPont member explained, the aim of using information from actual production processes was that:

We had to have some real things to build on, otherwise it would become too abstract.

To have access to information about the production processes, EDF had to promise secrecy, and sign a non-disclosure agreement. Creating this protected space implied that other chemical companies or NGOs could not join the discussions, even if they wanted to.

Thus, whereas Dewey might envisage all those involved in indeterminacy to participate in a joint inquiry, in the case of DuPont and EDF the interaction processes were not public. However, the outcome; the risk framework, did become public with a special website developed to this end (www.nanoriskframework.com). In response to the launch of the framework, an international coalition of more than twenty CSOs published an open letter in which they rejected the partnership as well as the risk framework as ‘fundamentally flawed’ (Civil society-Labor Coalition 2007). Thus, while a protected space for interaction might differ from Dewey’s perception of a reflective inquiry, the friction and tensions that emerged when the outcomes of the partnership between DuPont and EDF became publicly available provided insight into what is at stake and for whom. This, in turn, resembles the aim of reflective inquiry.

## Interactivity Within a Deweyan Public on Emerging Technologies

Dewey seems to assume that as soon as those who are involved in indeterminacy come together, they start to share their experiences, question each other, and articulate problems and ways to move forward. Previous research already showed that interactivity does not automatically occur when technology developers and civil society actors meet and discuss emerging technologies. As for example Hanssen (2009) and Te Molder (2011) showed, a common interaction pattern is actors voicing claims and statements on new technologies without questioning each other. This pattern became also visible in the NanoCap project. As part of the project, the NanoCap partners organized a closing event at the European Parliament in Brussels to present the position papers developed by the NGOs and trade unions. The aim of this event was to stimulate discussion with science and industry, in particular with regard to the issues of workplace safety and implementing the precautionary approach (see www.NanoCap.eu for more details). Although these issues are also a concern for technology developers, and representatives from science, industry, employers’ organizations were present, there was little response to the issues that were presented by the NGOs and trade unions. Generally, items from the position papers were only taken up as matters of common concern for all participants when a moderator identified them as such. For example during a panel discussion in which NanoCap members and representatives from industry participated, an EC policy officer who moderated the session, mentioned indeterminacy with regard to assessing the risks of nanoparticles: ‘What does it mean for legislation in Europe that there are no risk assessment methods for nanoparticles?’, and ‘Once we have assessed the risks, how do we decide whether these risks are acceptable or not?’ These questions could be considered a matter of concern for both CSOs and nanotechnology developers; for CSOs because they addressed these items in their position papers, and for industrialists because legislation and risk assessment are an integral part of the context within which they work. When asked by the moderator, participants in the panel discussion did articulate some dilemmas and expectations, but rarely responded to each other’s visions and positions and did not question each other.

This example from the NanoCap project shows that interactivity can be stimulated by explicit moderation. Also in CTA workshops, interactivity was stimulated by explicit moderation, as well as by using socio-technical scenarios as external input to start the discussion. These scenarios were developed upon ‘controlled speculation’ (Te Kulve and Rip 2011), starting with the actual dilemmas, concerns and activities of the actors involved as identified by the CTA analyst. An indeterminate situation gives, in principle, rise to different responses. These possible responses were the starting point for developing narratives that explored action-reaction patterns and the consequences of various possible actions. Such a scenario could thus be seen as a virtual reflective inquiry, providing substance to phases 1–3 of Dewey’s reflective inquiry. When reading the scenario, those who are involved can see themselves embedded in a broader development, and the narrative demonstrates how possible actions, interactions, and repercussions might play out in practice. In the workshop, scenarios functioned as a platform for actors to articulate problematic issues and develop and rehearse ways to deal with it, for example through developing new or adapted norms, values, roles and responsibilities (Parandian 2012; Krabbenborg 2013a).

The partnership between DuPont and EDF showed that joint product development is another way to stimulate interactivity. As the aim of DuPont and EDF was to develop a joint risk framework, they had to reach a consensus about what should be in the framework. DuPont staff and the project leader of EDF pointed out that there were differences of opinion between the partners on some issues, but nevertheless, as a DuPont staff member recalled:

We had a 360 degree look (…) we kept spinning around and everybody would look at it from their perspective.

What each of the four cases also made apparent is that inquiry into emerging nanotechnology and its societal impacts might require a somewhat protected space. We already saw that for DuPont and EDF a protected space was necessary because of the confidentiality of the information. The NanoCap project functioned as a protected space for interaction among the participants. As nanotechnology was a new topic for most participants, they wanted to freely explore the topic and develop their opinion without the intrusion of other actors, such as social scientists observing the project meetings.

## The Content of Inquiries into Emerging Technologies

With regard to the content of discussions, Dewey is optimistic in that he seems to assume that every type of indeterminacy will be recognized and settled somehow (see also Marres 2007, p. 768) so in his view no ‘orphan issues’ will remain. In our current society, however, research already demonstrates that risk issues, such as toxicology issues, prevail against other societal issues, such as ethical aspects and changing norms and values (Rip and Talma^1998^; Swierstra and Rip 2007). Risks of nanotechnology was also the main topic for discussion in the Dutch Societal Dialogue, while the initial aim of the Societal Dialogue was to focus on broader ethical and societal aspects, next to risk issues (Instellingsbesluit 2009; CIEMD 2009).

The fact that risk issues prevailed in the Dutch Societal Dialogue is not surprising given the fact that an entrenched cultural repertoire is available for these items that actors can fall back on. A repertoire functions as a toolkit and provides actors with a historically transmitted and publicly available system of symbols, myths, world-views, and stereotypes from which actors can draw certain elements to make sense of particular situations and shape their actions (Swidler 1986). In our Western societies, risk and privacy issues related to new science and technology can become a topic for deliberation relatively easily because by now, there is a toolkit available for these issues from which actors can (strategically) pick items. There are examples from earlier technologies that can be mobilized by participants in public engagement events. Moreover, there are professional institutions that have mandates and responsibilities to study the toxicity of chemicals, including nanoparticles, and/or inform citizens about health risks of nano-enabled products.^3^ For other societal issues, such as the way technology shapes how we relate to the world and to each other, and how it might change the way we value certain behaviours and norms (Boenink et al. 2010; Feenberg 1999; Swierstra and Te Molder 2012), there is much less of a repertoire available for actors to use as a toolkit. This leads to these types of issues remaining ‘orphans’, not taken up as topics for deliberation (Swierstra and Rip 2007).

But broadening of the debate can also occur. Again, there is a role for third persons. As part of the Dutch Societal Dialogue for example, several project organizers developed tools to stimulate discussion and elicit responses with regard to the broader ethical and societal aspects involved. Philosophers from the University of Twente in this regard developed vignettes on how specific nano-enabled devices might influence and change current societal values, norms, and habits in concrete situations, such as in the healthcare or food industries. And researchers from Eindhoven University developed future nano-consumer products to elicit responses on what people value in a product, why they want to buy certain things or not (www.nanopodium.nl).

## Effects in the Real World?

Did the inquiries between nanotechnology developers and civil society actors also lead to better informed, that is more encompassing decision making on NEST? (cf. phase 4 of Dewey’s reflective inquiry).

Not immediately. First, characteristic of the development and embedding of emerging technologies in our society is the fact that decisions are made throughout the innovation trajectory, and in different contexts, such as laboratories, firms, and government agencies. To be able to make informed decisions based on the input of reflective inquiries, outcomes of should ideally be aggregated and disseminated in the public sphere, the open space in society that is supported through a diffuse infrastructure that enables people to engage in extended deliberation (reaching across space and time) through a variety of media (Taylor 2002). In this way, other actors, not being part of the actual inquiry, can become aware of and take the outcomes into consideration. The case studies showed that aggregation and dissemination does not occur automatically, but that special efforts are needed. For example, NanoCap partners and DuPont and EDF created a special website to disseminate results; publicly available reports were developed in the case of the Dutch Societal Dialogue. The need to aggregate and disseminate outcomes also introduces another issue, namely whose issues and concerns are taken up in the formulation of overall outcomes? The case of the Dutch Societal Dialogue illustrated the phenomenon of selective aggregation. While a variety of issues, stakes and concerns were articulated during the actual interaction events, the final report that presented the results of the Societal Dialogue, mentioned only a small part of these issues (CIEMD 2011). A contributing factor to selective aggregation in this case was the fact that the Committee who had the responsibility to write the final report, turned out to be primarily interested in the process of dialogue, e.g. how many citizens were involved in the actual events, and not so much in the content of the actual debates (see Krabbenborg and Mulder 2015).

Besides, achieving better-informed decision making is not only a matter of creating actual interaction events, but also depends on whether and how the institutions where actors work (e.g. universities, companies, government agencies) have openings to take the new inputs into account. Follow up evaluations to assess the impacts of CTA workshops (cf. phase 4 of a Deweyan reflective inquiry) for example revealed that while participants learned from the workshops (Parandian 2012; Van Merkerk 2007; Krabbenborg 2013a, b), only a few participants experimented with the new insights acquired. As for instance Rip and Shelley-Egan (2010), Wynne (2007) and Nowotny (2007) already argued, there exists a discrepancy between individual learning and institutional learning. When participants leave the workshop and go back to their own institutions, they are confronted with existing rules, demands, and regulations that function in their research group or sector, for instance with regard to doing ‘good science’ or ‘being competitive at the market’. These rules and regulations at the institutional level shape the room for manoeuvre of actors. Thus to take up insights acquired from reflective inquiries in daily practices, there must also be openings, and flexibility, at the level of institutions.

## In Conclusion

Inspired by Dewey, I have linked the productive involvement of civil society actors with the need to address indeterminacies rather than merely involving civil society actors as new dialogue partners because they a priori represent ‘societal issues and needs’. For Dewey, public involvement always occurs in relation to an indeterminate situation. There is no public beforehand with which to engage. Instead, publics have to be searched for time and again, depending on what is at stake.

From the perspective of Dewey, it can be argued that the challenge we face is not to have a priori participation of civil society actors in research and innovation trajectories as seems suggested nowadays with the discourses of inclusive, deliberative governance (Commission of the European Communities 2001) and Responsible Research and Innovation (European Commission 2014; Owen et al. 2013). Instead, the challenge is how the involvement of civil society actors can improve the identification and articulation of emerging indeterminacies occasioned by the development of NEST. As I noted already in my PhD thesis (Krabbenborg 2013a), who should be involved and what types of interaction are productive both depend on the diagnosis of indeterminacy, and remain part of what has to be found out and articulated. This implies that not just any scientist, or, for that matter, any individual citizen or CSO, has to participate. A simple example is how scientists operating in the domain of nano-electronics will not have to participate in a reflective inquiry because no important real-time indeterminacies exist in their domain, while scientists operating in the domain of nano-enabled drug delivery for example, or nanotechnology and food have the responsibility to participate in reflective inquiries because indeterminacies exist. Of course, the situation is more complex, and is evolving, but it illustrates my point that the need to engage differs.

Dewey’s general idea of assemblages of publics that appear and disappear when the issues of concern have been addressed may not reflect what is happening in real life. In our society for example, there are professional spokespersons and official representatives for particular issues, such as dedicated NGOs, but also other type of civil society organizations (CSOs) who are seen as representatives of ‘societal needs and issues’. They can play a role in deliberation processes on the development of emerging technologies, but, speaking with Dewey in mind, having NGOs or CSOs as dialogue partners should not mean that the search for those who are affected comes to an end. It is easy to invite dedicated NGOs and CSOs to interaction events because of their visibility and their standing on particular issues. This is, as it were, engagement-supply push. My argument is that issue pull, as part of an inquiry into indeterminacies, must be the starting point (see also Marres 2005, 2007). This might well identify certain NGOs and CSOs as relevant participants in the space for interaction that is organized and/or emerging, but it also implies that those who are or might be affected might not be immediately visible and must be discovered and articulated.

The cases presented showed enabling and constraining conditions with regard to reflective inquiry in the twenty-first century. What we saw is that empirically informed sociotechnical analysis (e.g. in the form of scenarios), imagination, anticipation, and third persons are conducive to have ‘Deweyan publics’ and start a reflective inquiry into indeterminacies occasioned by new technologies (cf. also Lindblom 1990).

In closing, I note that not only direct interactions between technology developers and civil society actors have to be productive to address the open-endedness of emerging technologies, but existing institutions and their mandates/responsibilities to develop and embed newly emerging science and technology have to evolve as well to allow inquiry and deliberation among different relevant actors. In fact, there is a challenge for institutions and institutional arrangements to do justice to the dynamic and indeterminate character of emerging technologies. They should have a measure of flexibility so as to be able to respond to changes and accept tentative approaches, rather than strict arrangements. Thus the issue addressed in this paper of how to have more productive involvement of civil society actors in the development of emerging technologies and its embedding in society is not a matter of better interaction per se, but touches on foundational issues related to the functioning of our society. The ongoing effort to create upstream public engagement, now also for other emerging technologies, like synthetic biology and geo-engineering, can be taken up as an opportunity to shape our society more reflexively.

## Footnotes

1 It is important to note that for Dewey reflective inquiry refers to an ideal situation rather than something that already occurs in real life. One of the problems he observed for instance is that people in his era were oriented to consumption and exposed to a variety of entertainment. Direct satisfaction became more important according to Dewey than inquiry and the articulation of public affairs (Dewey 1927).

2 In Dewey’s pragmatist philosophy, an important aim is to cultivate and expand the capacity of human beings to intelligently navigate a world in flux and to solve actual problems as these are experienced in everyday life. In developing his philosophy, Dewey is concerned with the question of how to improve relations between the interpretive frameworks of human beings, that is, their perspectives, values, institutions, and beliefs, and a continually changing environment. The ideal relation would be that a human being is able to solve actual problems and modify their environment and thus not become a victim of change.

3 In the Netherlands, RIVM, the public research institute for public health and environment, would be an example. See www.rivm.nl/Onderwerpen/Onderwerpen/N/Nanotechnologie/Kennis_en_informatiepunt_risico_s_KIR_Nanotechnologie.
